# Bearing Capacity Performance and Optimal Design of a Novel Aluminum Alloy Arch Gusset Joint

**DOI:** 10.3390/ma16227165

**Published:** 2023-11-15

**Authors:** Huaihao Wang, Rongxin Guo, Yubo Zhang, Yang Yang, Weirong Xiao

**Affiliations:** 1 Yunnan Key Laboratory of Disaster Reduction in Civil Engineering, Faculty of Civil Engineering and Mechanics, Kunming University of Science and Technology, Kunming 650500, China; whh1208bb@icloud.com (H.W.); guorx@kmust.edu.cn (R.G.); yangyang0416@kmust.edu.cn (Y.Y.); weirongxiao@kust.edu (W.X.); 2 Faculty of Architecture and Civil Engineering, Yunnan Agricultural University, Kunming 650000, China

**Keywords:** aluminum alloy gusset joint, planar angle, static bearing performance, failure mode, numerical analysis

## Abstract

Current research on aluminum alloy gusset joints has neglected the influences of the angle between members and the curvature of the joint plate on joint performance. This study introduces the concept of the planar angle and establishes 16 joint models using ABAQUS finite element software with parameters such as the planar angle, arch angles, joint plate thickness, web thickness, and flange thickness. The load-bearing capacity of the novel aluminum alloy arch gusset joint is theoretically analyzed, and the concepts of strong and weak axes are proposed. The failure modes and significance of different parameters regarding the bearing capacity and initial stiffness of the joint under various parameters are summarized. The results indicate that the planar and arch angles significantly affect the bearing capacity, stiffness, and failure mode of the joint.

## 1. Introduction

Aluminum is the most abundant metal in the Earth’s crust, and aluminum alloys are widely used in the construction industry owing to their low density, high strength, and corrosion resistance. They are particularly suitable for large-span spatial structures, such as lattice shell structures. The design and use of lattice-shell structures have developed rapidly since their inception in 1863. The “Explorer” Dome, built in the UK in 1951, is the oldest aluminum alloy lattice shell structure in the world [1]. With technological advancements and improvements in processes, aluminum alloy space grid structures have been widely applied in various applications, such as sports stadiums, theaters, and chemical storage tanks. Examples include the Schiphol Airport Aviation Museum, completed in 1971; the C.B.R. Cement Company limestone storage silo, completed in 1990; the Henry Doorly Zoo desert dome, completed in 2002; and the Nanjing Foding Palace, constructed in 2015. Aluminum alloy space grid structures are divided into three main types: embedded hub, bolted ball, and gusset joints [2]. Gusset joints are composed of upper and lower joint plates that connect several members. Most lattice shells use bolted connections for joints, with a few using bolted connections.

In recent years, many researchers have analyzed aluminum-alloy gusset joints by theoretical analysis, numerical simulations, and experimental studies. Since the 1970s, the scientists in the United States and Europe have conducted research on and analyses of aluminum alloy structures. Shibata et al. [3] obtained the moment–rotation curves of two types of joints through experiments and simulations, and they showed that the bearing capacity of lattice shell structures increased with increasing joint stiffness. Chenaghlou obtained the moment–rotation curves of joints under different conditions by changing the axial force and bending moment on the joints. Kato et al. [4,5] conducted bidirectional and tridirectional limit-bearing capacity experiments on lattice shells with initial defects and showed that the stiffness has a greater impact on the limit-bearing capacity than the initial defects. López et al. [6,7] conducted research on the nonlinear behavior of single-layer domes by changing their geometric parameters and joint stiffness, proposed an ORTZ (spherical node connection system) joint model for estimating the bearing capacity of lattice shells, and experimentally verified the reliability of the formula. Over the past decade, with the rapid development of Chinese cities and the constant evolution of housing structures, the superior performance of aluminum alloy structures has become increasingly apparent. A large number of researchers have conducted in-depth studies of aluminum alloy gusset joints, as they are applied in more practical engineering projects. From 2015 to 2016, Guo et al. [8,9,10,11] conducted axial compression tests on 63 Chinese-made 6061T6 aluminum alloy profiles and verified the relevant specifications. They summarized the failure modes of AAG (aluminum alloy gusset) joints through experiments and simulations, proposed a formula for the bending stiffness of AAG joints, and recommended calculation formulas for the tearing resistance and local buckling resistance of AAG joint plates. Convenient design guidelines were provided to avoid block tearing and local buckling of AAG joint plates. In 2017, Zhe et al. [12,13,14] conducted parametric studies of the buckling performance of shells and proposed a theoretical formula to predict the elastoplastic buckling load of AAG joint shells. They studied the effects of component height, axial force, bolt pre-tension, bolt clearance, and material nonlinearity on the bending performance of AAG joints. In 2018, Guo and others [15,16] studied the failure modes of aluminum alloy joint plates at high temperatures and proposed theoretical formulas that can estimate the bearing capacity and bending stiffness of AAG joints at 300 °C. Since 2019, most domestic researchers have focused on aluminum alloy joints. Liu [17,18,19] proposed a high-temperature reduction factor for the ultimate bearing capacity of Al alloy joints. They combined numerical simulations with experiments to propose an effective method for predicting crack locations. Wang et al. [20,21] proposed a new type of aluminum alloy joint (FGC joint). Research has shown that the bearing capacity, shear stiffness, and bending stiffness have been improved accordingly, and a change in the joint plate thickness significantly affects the bending stiffness and bearing capacity of the joint. Huihuan et al. [22] also proposed a new type of joint, an aluminum alloy hollow angular plate joint (AHP), and extracted its bending moment–transfer curves under in-plane and out-of-plane bending moments. The results indicated that the bending stiffness and bearing capacity of the AHP joints were significantly improved. Sugizaki et al. [23,24] analyzed the rigidity of aluminum alloy single-layer spherical grid shell embedded joints. They conducted compression and bending tests of the primary nodes with triangular mesh elements, suggesting that these joints exhibit out-of-plane rigidity and in-plane semi-rigidity. Hiyama et al. [25] performed numerical simulations and experimental analyses of single-layer grid shell bolted spherical joints. They proposed formulas for joint stiffness and bearing capacity, noting that the increases in joint stiffness and bearing capacity are directly proportional to the cross-sectional area. Kato et al. [4,5,26] conducted nonlinear elastic-plastic hinge analyses of three-dimensional beam–column members with elastic-plastic hinges at both ends and the midspan. They discussed the buckling failure of semi-rigid ball joints in steel grid shells and their analysis methods. Additionally, they analyzed the factors influencing the overall stability of the grid shell.

In summary, there have been some shortcomings in the research on aluminum alloy plate joints, including the following: (1) in some finite element analysis models, the preload of bolts has not been considered, and in some models, only node coupling has been considered without creating a solid model of the bolts; and (2) most finite element analysis models have ignored the effect of in-plane bending moments, whereas in reality, the in-plane bending stiffness of plate joints is much smaller than the out-of-plane bending stiffness, so the rationality of this simplification assumption requires further research.

Owing to their excellent performance, aluminum-alloy gusset joints have been widely used in large-span building structures. However, the previous studies still lacked the following content: with the diversification of building structures, individualization of building appearance, and inevitable changes in joint construction forms during construction, the structural design of aluminum alloy gusset joints has changed. (1) Existing research on aluminum alloy gusset joints assumes that the angle between members is equal, mostly 90° or 60°; and (2) the joint plates are mostly flat. However, in actual projects, such joints often cannot meet construction and structural requirements. In this study, ABAQUS2020 finite element software was used to model actual projects combined with orthogonal experiments, and the concept of the planar angle was proposed for the first time. The effects of the member angle (planar angle), joint plate curvature (arch angles), joint plate thickness, web thickness, and flange thickness on the bending performance of the new type of aluminum alloy plate joint were analyzed. By combining mechanical theory analysis, the concepts of the strong and weak axes of the joint plate are proposed, and the failure modes, force transmission mechanism, bending moment-transfer curves, and initial stiffness curves of the new type of aluminum alloy plate joint are discussed. This study provides guidance and suggestions for applications in future research.

## 2. Experimental Verification of Finite Element Model

### 2.1. Experimental Model

**Figure 1:**
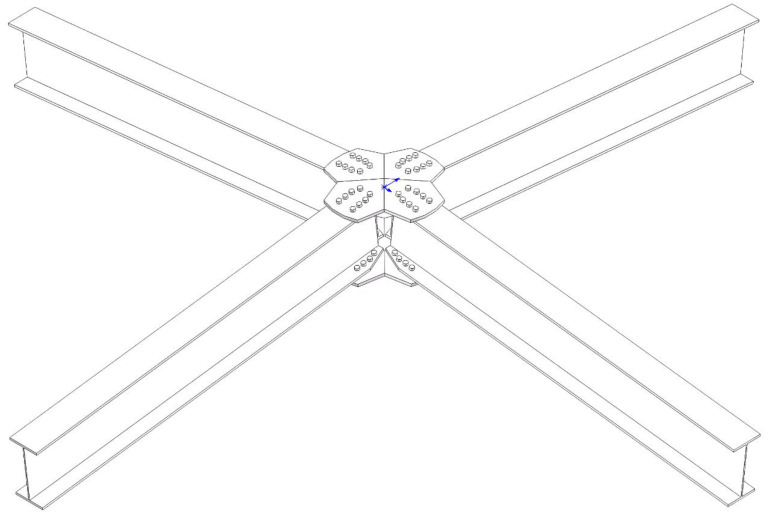
Gusset Joint Schematic Diagram.

**Figure 2:**
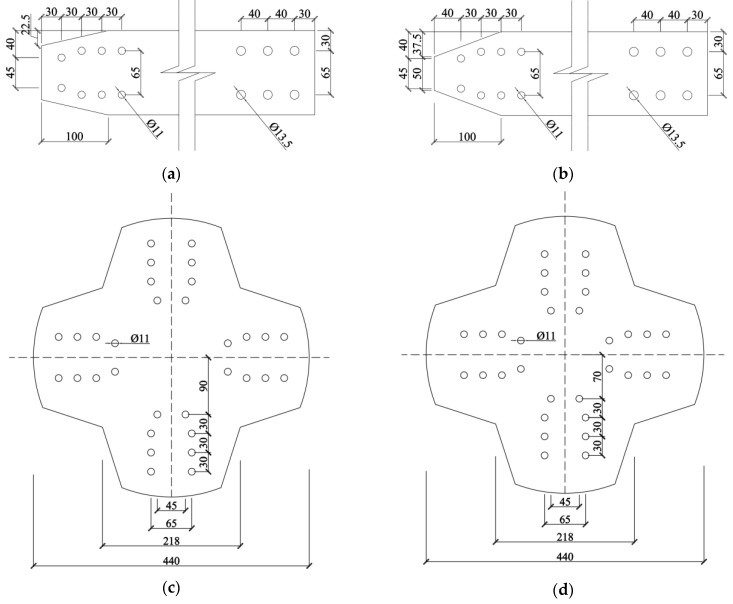

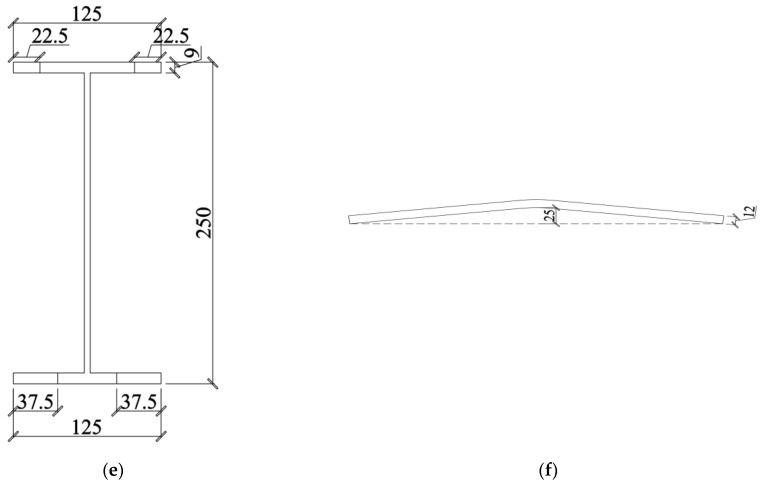
Gusset Joint Dimension Schematic. (**a**) Details of upper flange (**b**) Details of lower flange (**c**) Details of upper gusset plate (**d**) Details of lower gusset plate (**e**) Details of member section (**f**) Arching of gusset plate.

The verification model in this study was adopted from the research of Wu et al. on the static performance of arched joints [27]. An arched joint experiment in the literature was simulated using ABAQUS, and the results were compared with the experimental results to verify the accuracy and reliability of the finite element simulation analysis, thereby providing a technical basis for the subsequent numerical analysis. The joint system consisted of four H-shaped members and two joint plates at the top and bottom. The joint plates were connected to the members using bolts, and the joints were arched at a rise angle of 7°. The H-shaped members had a cross-sectional size of 250 × 125 × 5 × 9 mm, length of 1650 mm, and an angle of 90° between adjacent members. The bolt diameter was 10 mm with 16 bolts on each member. An overall schematic of the joint is shown in Figure 1, In the diagram, the blue arrow indicates the reference coordinate system used during modeling, and the detailed parameters are shown in Figure 2. The material properties were obtained through aluminum alloy material tests. The elastic modulus, nominal yield strength, and tensile strength of the aluminum alloy were 70,400 MPa, 264 MPa, and 304 MPa, respectively. The elastic modulus, nominal yield strength, and tensile strength of the stainless-steel bolts were 189,000 MPa, 460 MPa, and 720 MPa, respectively. In the finite element validation modeling process, all parameter settings aligned with the experimental conditions reported in reference [27], and the modeling process followed the method presented in reference [27]. Solid elements (C3D8R) were used for all components in the model. Four contact pairs were defined, including a bolted plate contact, a nut component contact, a connecting plate flange contact, and a bolt rod-bolt hole contact. The analysis was divided into five steps for improved convergence. In the initial analysis step, essential boundary conditions were applied to the connecting plates, components, and other relevant parts. In the second step, all degrees of freedom of the components were temporarily restricted, and a small force (100 N) was applied to the bolts to establish contact between the bolts and the components and connecting plates. In the third step, the temporary constraints on the component degrees of freedom were removed. In the fourth step, the pretension force was applied to the bolts, and the amplitude was adjusted to “smooth,” resulting in a smooth pretension force of 24.6 kN in the bolts. In the fifth step, displacement loads were applied to the connecting plates. The boundary conditions were set to mimic the experimental setup, with all four rods allowed to hinge, accurately replicating the real experimental conditions.

### 2.2. Simulation Verification

**Figure 3:**
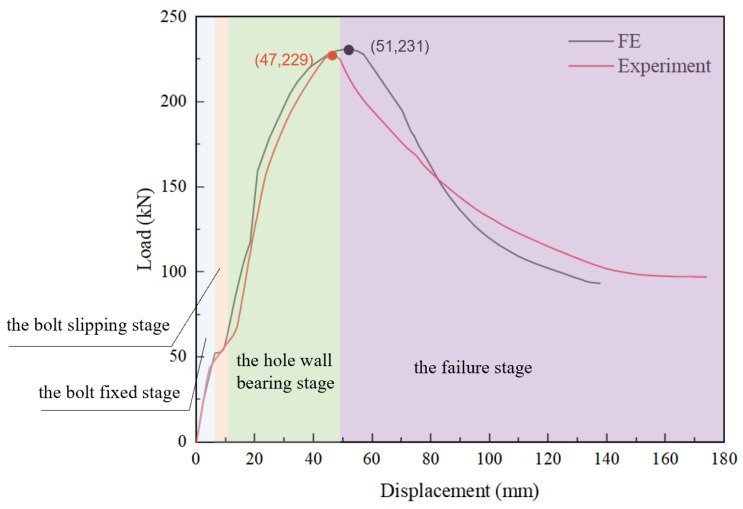
Comparison of Load–Displacement Curves.

**Figure 4:**
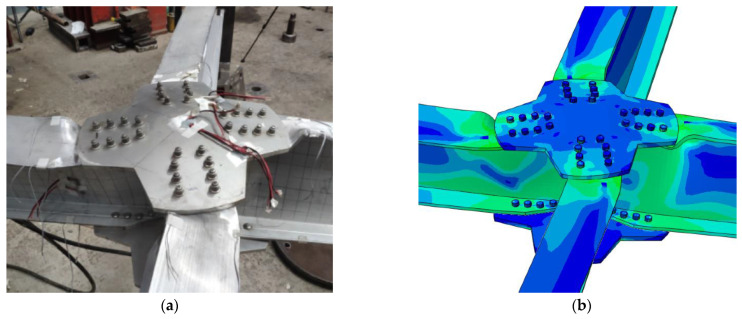
Comparison of Failure Model. (**a**) Final Failure Mode of Test, Reproduced with the Permission of [27], Copyright @Elsevier. (**b**) Final Failure Mode of FE Model.

During the finite element verification, all parameter settings were consistent with the experimental conditions reported in the literature [27]. The vertical displacement and vertical reaction force at the loading point were extracted from the finite element simulation results and compared with the experimental results by Wu et al., as shown in Figure 3. It can be observed that both the load-displacement curves obtained from the finite element simulation and the experiments exhibited four distinct stages, namely the bolt fixed stage, the bolt slipping stage, the hole wall bearing stage and the failure stage. During the bolt fixed stage and the hole wall bearing stage, the curves from the finite element simulation and experiments were almost identical. Finite element simulation and experiments showed a difference of only 1.34% in initial stiffness. As the load increased, the component entered the plastic phase until it reached the ultimate load, with a difference of only 0.87% from the experimental results for the ultimate load. The unique failure mode corresponding to the ultimate load had a difference of only 8.5% from the experimental results. The main reasons for the disparity were primarily incomplete alignment between the boundary conditions used in the finite element analysis and the real experimental setup. Additionally, there might have been certain material processing defects and some errors in the installation of test specimens during the experimental phase. Further comparison of the failure modes between the finite element and experimental results showed that both had the same failure mode, namely local buckling of the web and bending-torsion failure of the components, as shown in Figure 4. The finite element simulation had a high degree of agreement with the experiment, thereby verifying the accuracy of the finite element model establishment and analysis in this study.

## 3. Orthogonal Experimental Design

Orthogonal experimental design is a comprehensive and systematic experimental design method widely used in industrial and scientific research. Through an orthogonal experimental design, different combinations of various factors can be reasonably allocated to obtain more extensive experimental data, thereby improving experimental efficiency and reducing experimental costs.

Wang et al. [28] showed that the main geometric parameters affecting the bending capacity and initial stiffness of aluminum alloy gusset joints are the joint plate thickness ($t_{p}$), web thickness ($t_{w}$), and flange thickness ($t_{f}$). However, traditional research has neglected the influences of the angle between the members (planar angle, α) and curvature of the joint plate (arch angle, β) on the bending capacity and initial stiffness of the joints. This study focused on an orthogonal experimental design for these five parameters. The planar and arch angles were determined based on the actual engineering background of this study. Wu et al. [27] studied the influence of the arching angle on joint performance. Their study showed that the ultimate bearing capacity and initial stiffness increased with the arching angle of the joint. However, in Wu et al.’s study, the angles between the members were the same, and the influences of changes in joint plate thickness, web thickness, and flange thickness on the arched joint’s performance were not discussed. This paper aims to further explore the impact of these five factors ($t_{p}$, $t_{w}$, $t_{f}$, α, β) on the new aluminum alloy plate joint, As shown in Figure 1, this paper has designed an orthogonal test table with five factors and four levels (Table 1). However, due to the presence of five factors, conducting studies while controlling a single variable would require a considerable number of simulation analyses, making the study less efficient. Orthogonal experiments can effectively address this issue by providing information about the impacts of different levels and factors on response variables with a limited number of simulation analyses. In this paper, the response variables are the initial stiffness and the ultimate bearing capacity.

## 4. Finite Element Model Establishment

### 4.1. Geometric Parameters

**Figure 5:**
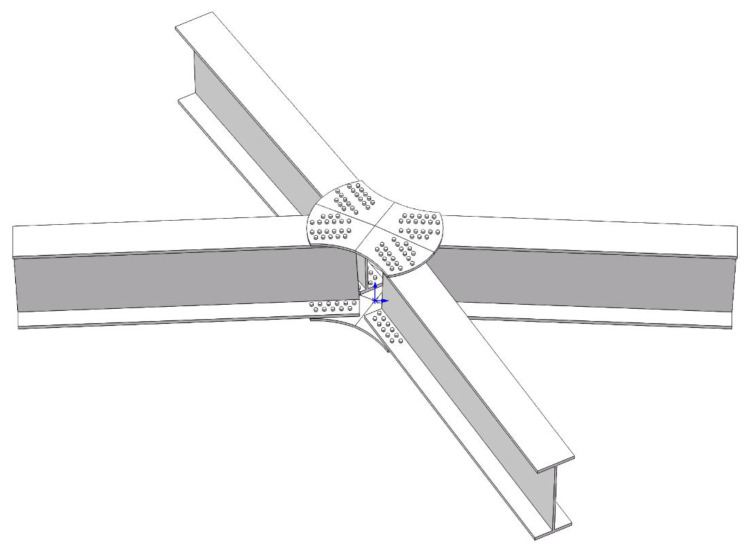
Schematic Diagram of Gusset Joint.

**Figure 6:**
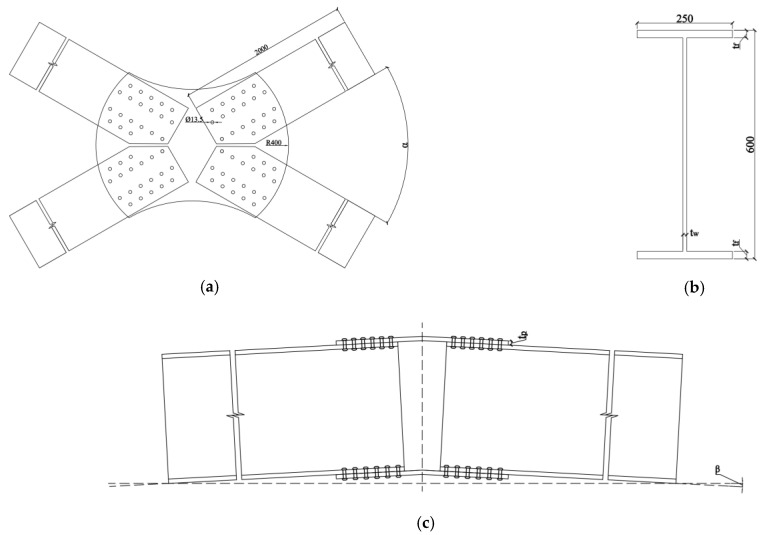
Details of Gusset Plates. (**a**) Top view of gusset joint. (**b**) Details of member section. (**c**) Front view of gusset joint.

The novel aluminum alloy arched gusset joint investigated in this study is shown in Figure 5 (overall component diagram), In the diagram, the blue arrow indicates the reference coordinate system used during modeling. The entire joint system consists of four aluminum alloy members and two joint plates at the top and bottom, where the joint plates are connected to the members by bolts. The member width, height, and length were 250, 600, and 2000 mm, respectively. The angles between the members were 40°, 50°, 60°, and 90° and the arch angles of the joint plates were 0°, 3°, 5°, and 7°. The number of bolts was calculated based on the most unfavorable working conditions in actual engineering, with 20 bolts on each upper and lower flange of a single member. The joint plate design changed according to changes in the angle between the members and the rise angle. The bolt rod diameter is 12.7 mm, and considering the gap between the bolt rod and the bolt hole after installation in actual engineering, the bolt hole diameter was set to 13.5 mm. The specific parameters of the joint are shown in Figure 6 (a detailed component diagram).

### 4.2. Material Model

**Figure 7:**
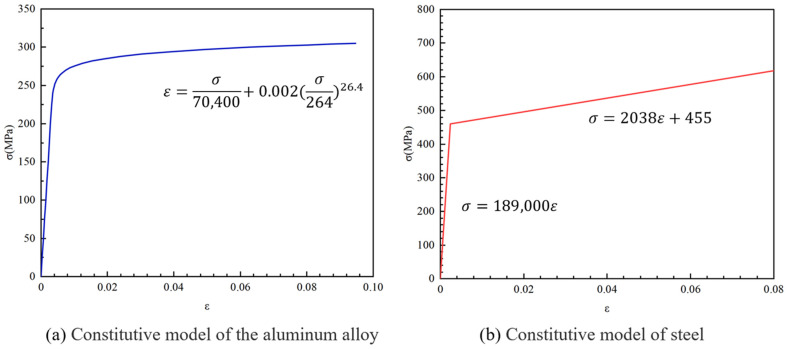
Constitutive Model of Materials.

The material properties of all components in this study were based on the results of performance experiments [28]. The tensile test results are shown in Table 2, where E represents the elastic modulus, $f_{0.2}$ represents the nominal yield strength, and $f_{u}$ represents the ultimate tensile strength. The Ramberg–Osgood model and Steinhard’s suggestion were used to simulate the material constitutive relationship of the aluminum alloy (Figure 7a), and a bilinear model was used to simulate the constitutive relationship of the bolts (Figure 7b). The Ramberg–Osgood model and Steinhard’s suggestion are currently the most commonly used models for representing the stress–strain relationship in Al alloys. Guo et al. [29] showed that the Ramberg–Osgood model can be used to describe the 6061T6 aluminum alloy produced in China, and n in the expression can be calculated using Steinhard’s suggestion. This expression is expressed by Equation (1), where E is the material elastic modulus, and n represents the strain-hardening exponent, which is usually calculated using Equation (2). For convenience, Steinhard proposed a simplified algorithm, as shown in Equation (3). Here, $\sigma_{0.1}$ is the stress value when the residual strain of the aluminum alloy is 0.1%, and $\sigma_{0.2}$ is the stress value when the residual strain of the aluminum alloy is 0.2%. It should be noted that ABAQUS2020 software requires the input of the true stress–strain relationship of the material for finite element modeling and calculation, whereas the engineering stress–strain relationship of the material obtained by simulating the material constitutive relationship of aluminum alloy using the Ramberg–Osgood model and Steinhard’s suggestion and simulating the constitutive relationship of bolts using the bilinear model can be converted into true stress and true strain using Equations (4)–(6). Here, $\sigma_{true}$ represents the true stress $\sigma_{eng}$ represents the engineering stress obtained in the tensile test, $\varepsilon_{true}$ represents the true strain, $\varepsilon_{eng}$ represents the engineering strain obtained in the tensile test, $\varepsilon_{pl,true}$ represents the true plastic strain, and E is the elastic modulus.

(1) $$\varepsilon =\frac{\sigma }{E}+0.002{\left(\frac{\sigma }{\sigma_{0.2}}\right)}^{n}$$

(2) $$ln(2)/ln(\sigma_{0.2}-\sigma_{0.1})$$

(3) $$n=\sigma_{0.2}/10$$

(4) $$\sigma_{true}=\sigma_{eng}(1+\varepsilon_{eng})$$

(5) $$\varepsilon_{true}=ln(1+\varepsilon_{eng})$$

(6) $$\varepsilon_{pl,true}=\varepsilon_{true}-\frac{\sigma_{true}}{E}$$

### 4.3. Element Settings and Mesh Division

**Figure 8:**
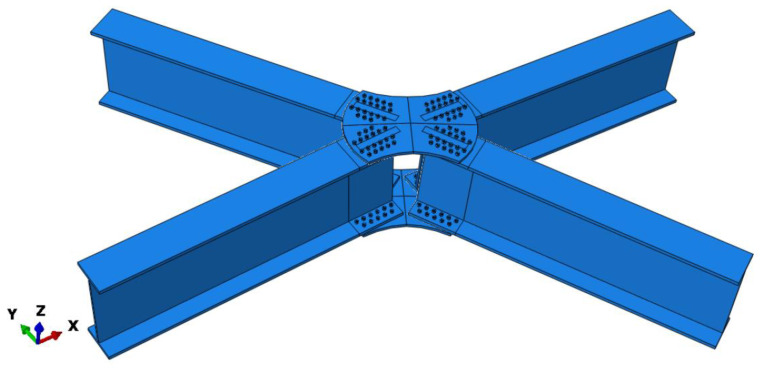
Full-scale Finite Element Model.

**Figure 9:**
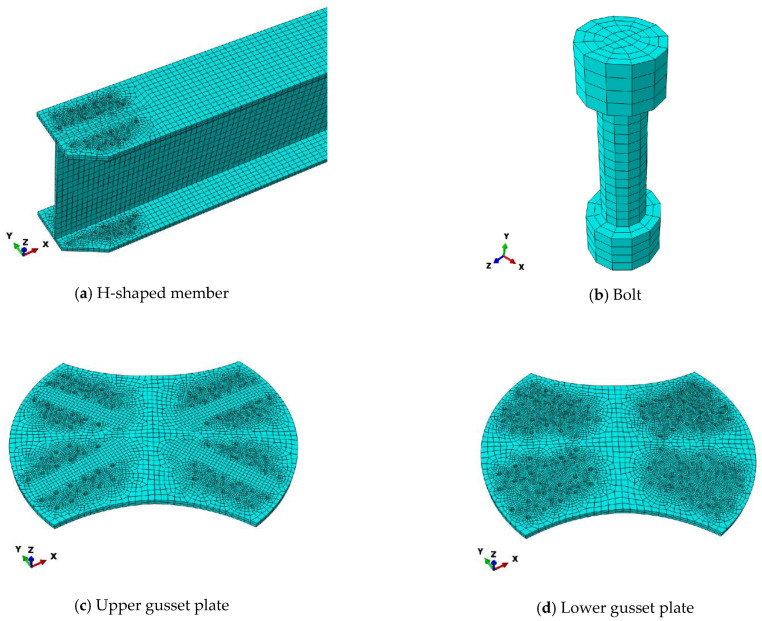
Component Mesh Division.

A finite element numerical model was established using ABAQUS2020based on the aforementioned geometric parameters and material model. Because the novel aluminum-alloy arched gusset joint is not a traditional joint, this study investigated joints with different angles between adjacent members. Therefore, a full-scale model was established using ABAQUS2020, as shown in Figure 8. In the full-scale model, a C3D8R element was used to model the joint plates, bolts, and members, as shown in Figure 9. This element is a typical eight-joint, fully integrated linear hexahedral element that can accurately calculate the stress and strain distributions of a structure. Because of the large number of joints, it can better adapt to the deformation of the structure and control the hourglass effect. The simple shape can improve the computational efficiency. The flanges and webs of the H-section components are divided into two layers of mesh along the thickness direction. The central part of the H-section component uses a relatively sparse mesh because the stress distribution in this part is uniform, and deformation is minimal. Due to stress concentration at the bolt holes, mesh refinement was performed, with a mesh size of 3 mm.

### 4.4. Contact and Analysis Steps

Contact settings are crucial for establishing the ABAQUS model. The contact settings affect the force transfer in finite element simulations, prevent object penetration, and establish a correct frictional force relationship. If the contact settings are incorrect, the simulation results may be distorted, thereby resulting in erroneous conclusions. In this study, there were four contact pairs in the model, including bolt–joint plate contact, nut–member contact, joint plate–flange contact, and bolt rod–bolt hole contact. In the contact pair settings, the master and slave surfaces were set based on several factors, such as material hardness, surface geometry, and contact type. In general, the master surface is harder with a larger mesh division. The detailed settings are listed in Table 3. In addition to the importance of contact settings in the model establishment, reasonable analysis step settings are equally important for the convergence of the model calculation. To make the model convergence easier, five analysis steps are defined throughout the calculation process: in the initial analysis step, the inherent boundary conditions are applied to the joint plate, member, and other related parts; in the second step, all degrees of freedom of the members are temporarily constrained, and a small force (100 N) is applied to the bolts to make contact between the bolts and the members and joint plates; in the third step, the temporary constraints on the members’ degrees of freedom are removed; in the fourth step, the bolt pre-tightening force is applied and the amplitude is adjusted to “smooth,” smoothly adjusting the pre-tightening force in the bolt to 24.6 kN; and in the fifth step, the displacement load is applied to the joint plate.

### 4.5. Boundary Conditions and Loading Scheme

**Figure 10:**
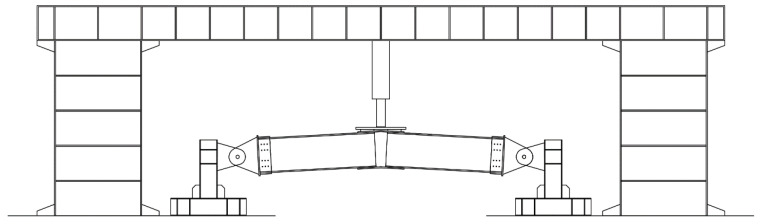
Loading Device Schematic.

**Figure 11:**
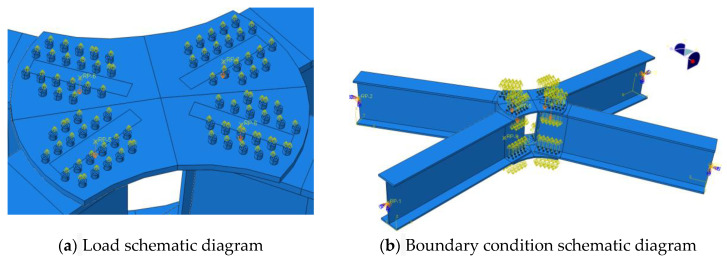
Load and Boundary Condition Schematic Diagram.

Because this study uses a full-scale model, the consideration of boundary conditions and the application of loads aim to reproduce the experimental scenario as much as possible. In the experiment, four members were connected to fixed hinge supports, and the loading device consisted of a portal reaction frame and a hydraulic jack. The hydraulic jack was connected to the center of the upper joint plate through a loading plate. The loading device is shown in Figure 10, with four loading blocks welded to the loading plate in contact with the upper joint plate. In the finite element simulation, the setup of loads and boundary conditions is illustrated in Figure 11; reference points were set at the ends of the four members, and the reference points were coupled with the ends of the members for motion coupling, releasing only the degrees of freedom along the strong axis to achieve a fixed hinge joint effect. In the upper joint plate, four loading areas were divided based on the experiment in the finite element model, and the reference points were coupled. Vertical displacement loads were applied at the reference points. The bolt pre-tightening force was 24,600 N, applied through the bolt force in the load options, and “fixed at the current length” was selected.

## 5. Theoretical Analysis

**Figure 12:**
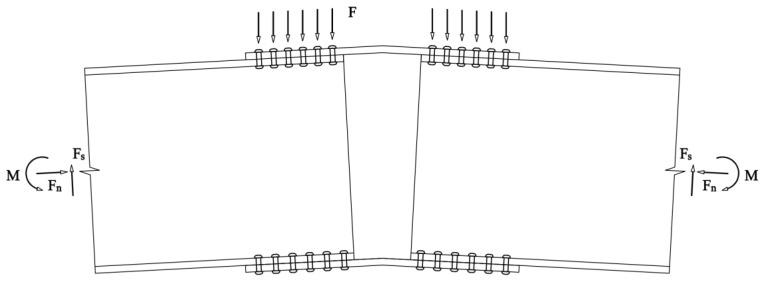
Force Analysis of Members.

**Figure 13:**
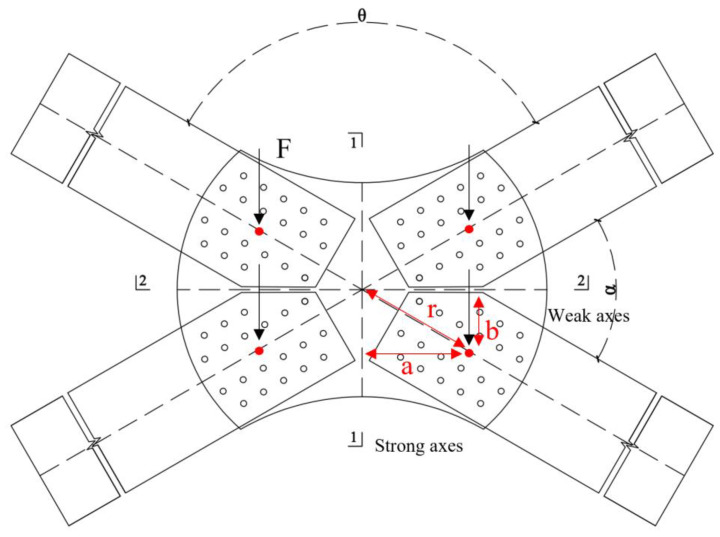
Force Analysis of Gusset Plates.

The most significant difference between the gusset joints investigated in this study and traditional joints is the change in the angle between the members, i.e., the change in the planar angle. The stress state of the joint plate changed significantly with changes in the planar angle. As shown in Figure 12, the joint system was primarily subjected to bending moments, axial forces, and shearing forces. In Figure 13, since the angles α and θ between the members are not equal, the distance a from the vertical load on the joint plate to axis 1-1 is greater than the distance b to axis 2-2. This difference caused the bending moment experienced by the section separated by symmetry axis 1-1 (referred to as section 1-1) to be greater than that experienced by the section separated by symmetry axis 2-2 (referred to as section 2-2). In Figure 13, four identical vertical concentrated forces F are applied at four symmetric points on the axis of the member. The bending moments borne by sections 1-1 and 2-2 are shown in Equations (7) and (8), respectively, where F is the concentrated force, a is the distance from the load application point to section 1-1, b is the distance from the load application point to section 2-2, and r is the distance from the load application point to the center of the joint plate. From the equations, it can be seen that, as the angle α between the members decreases, M_(1-1) increases, while M_(2-2) decreases. Owing to the change in the planar angle, the two symmetric sections of the joint plate experienced uneven stress and had unequal section moduli. This study introduces the concepts of strong and weak axes to further explain this phenomenon.

(7) $$M_{1-1}=2Fa=2Frcos(\frac{\alpha }{2})$$

(8) $$M_{2-2}=2Fb=2Frsin(\frac{\alpha }{2})$$

**Figure 14:**
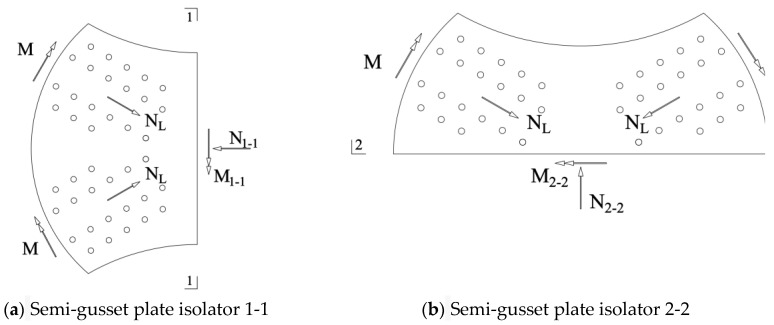
Force Analysis of Semi-Gusset Plate Isolators.

By dividing the joint plate into halves with two symmetry axes, an isolated body was obtained, as shown in Figure 14 where M is the bending moment transmitted from the member to the joint plate, M1-1 is the total bending moment in section 1-1 of the isolated body, M2-2 is the total bending moment in section 2-2 of the isolated body; NL is the axial force transmitted from the member to the joint plate, N1-1 is the total force on section 1-1 of the isolated body, and N2-2 is the total force on section 2-2 of the isolated body. In Figure 14, it is evident that the area of section 2-2 is larger than that of section 1-1, resulting in a greater section modulus for section 2-2 than for section 1-1. Bending moment MS2-2 experienced in section 2-2 was smaller than bending moment MS1-1 experienced in section 1-1, making section 1-1 more prone to failure. Section 1-1 is defined as the weak axis, and section 2-2 is defined as the strong axis. A change in the planar angle of the members leads to uneven stress and unequal section moduli in the two symmetric sections, rendering the joint more likely to rotate around the weak axes more prone to failure. In the following sections, we verify the validity of this theory using simulations.

## 6. Result Analysis

### 6.1. Failure Modes

According to Guo et al. [30], the failure modes of traditional gusset joints mainly include local buckling of the joint plate, net section failure at the end of the member, and plate-shaped tensile–shear failure of the joint plate. Wu et al. [28] found that the failure modes of flat gusset joints (with zero camber) were mostly net section failures of the members, whereas the failure modes of cambered gusset joints were mainly local buckling failures of the web. Their research showed that the camber angle had a significant impact on the stiffness, bearing capacity, and failure modes of aluminum alloy joints. However, for both traditional and cambered gusset joints, the angle between the members is fixed, mostly at 90° (four members) or 60° (six members), and most gusset joints are flat. In this study, combined with practical engineering, the planar and camber angles between the members were varied, and the resulting failure modes were mainly divided into three types: local buckling failure of the web, compression failure of the joint plate middle, and net section failure of the member.

As this section shows the changes in failure modes caused by changes in planar and arch angles, the naming in the figures only displays arch angles and planar angles, such as 3–40 representing an arch angle of 3° and a planar angle of 40°.

**Figure 15:**
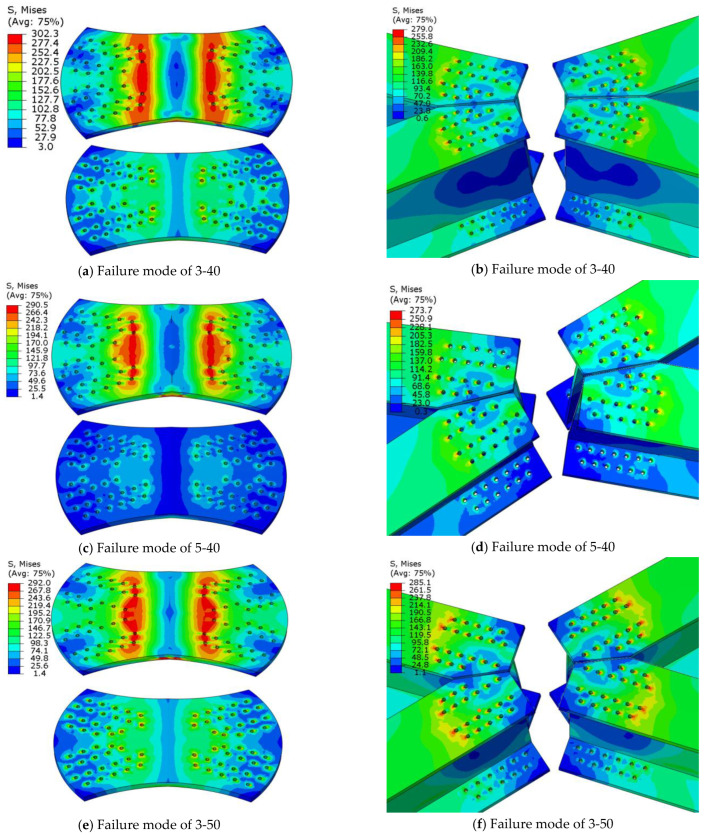

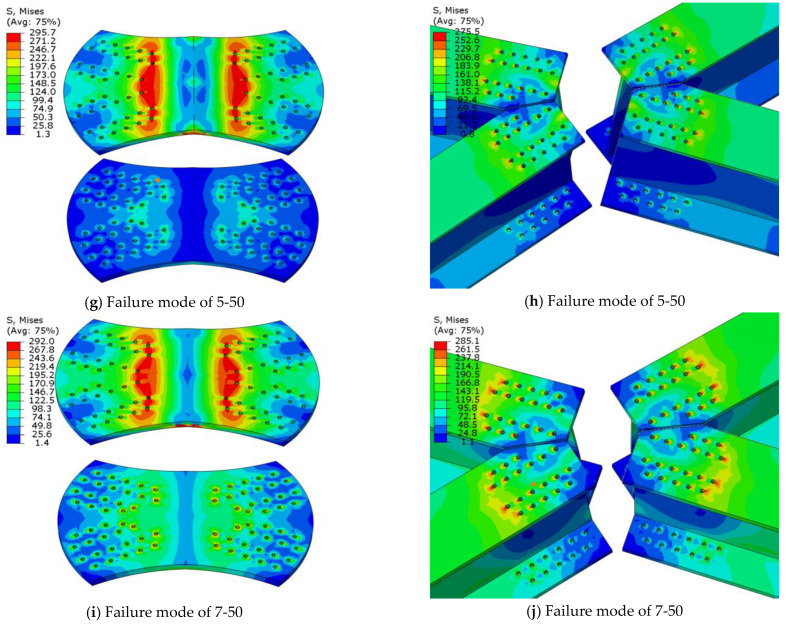
Compression Failure of the Joint Plate Middle.

The compression failure of the joint plate middle mainly occurs when the planar angles between adjacent members are unequal, and the joint plate has a camber angle, i.e., in the case of planar angles of 40°, 50°, and 60° and arch angles of 3°, 5°, and 7°, as shown in Figure 15. The failure mode was the compression failure of the joint plate middle, which was mainly due to uneven stress on the joint plate caused by the change in the angle of the members and the camber angle of the joint, leading to the overall compression of the component, with the entire joint bending and compression around the weak axis. Its cross-sectional bearing capacity against bending is weak, leading to a compression failure mode in the middle of the joint plate.

**Figure 16:**
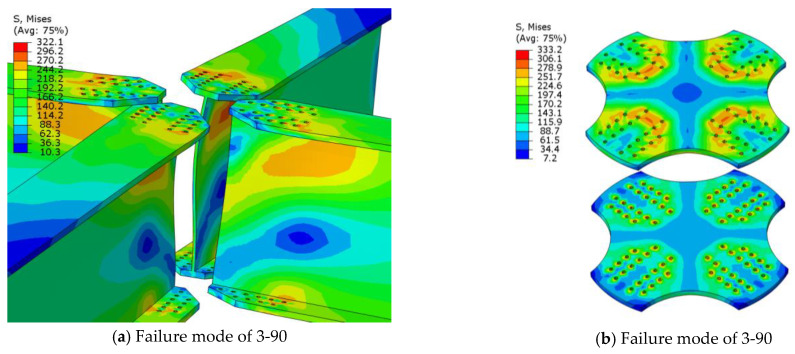

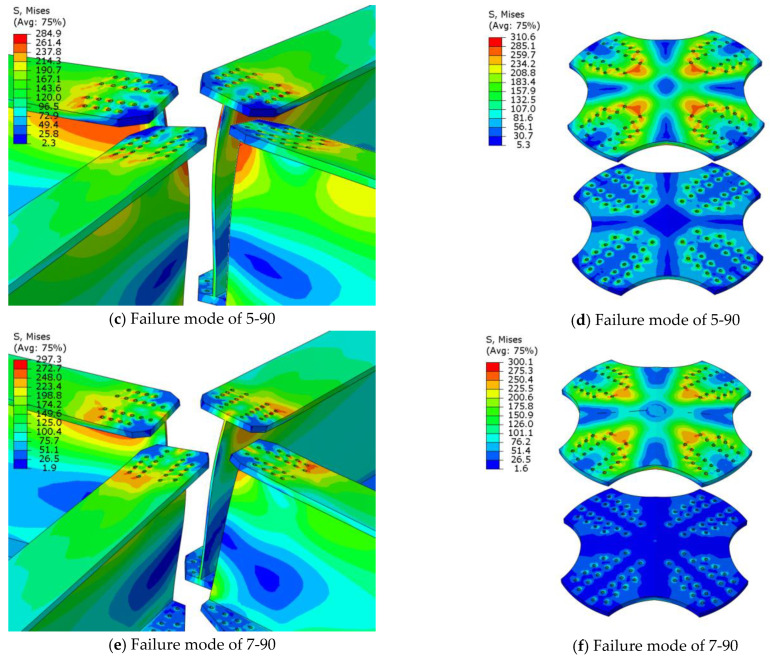
Local Buckling Failure of Web Plate.

The failure modes of joints with a member angle of 90° and an arch angle and joints with member angles of 40°, 50°, 60°, and an arch angle of 0° are local buckling failures of the web, as shown in Figure 16 This failure occurred mainly because the angles between the members were equal, the joint plate was subjected to uniform stress, both symmetric sections experienced equal stress, and the overall bending resistance of the joint plate was enhanced. The load was transferred to the members, causing local web buckling.

**Figure 17:**
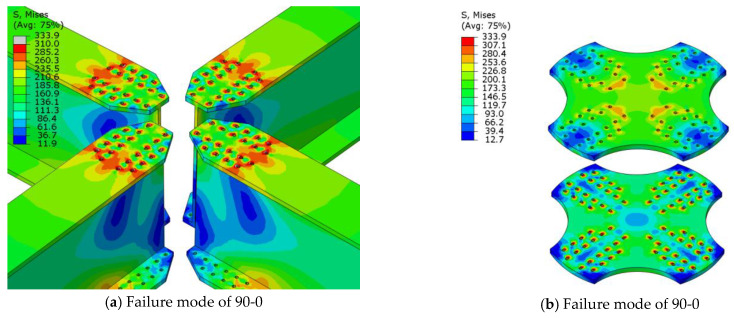
Net Cross-Section Failure of Members.

The failure mode of the joints with a member angle of 90° and arch angle of 0° was the net section failure of the member, as shown in Figure 17. In this case, the failure mode was the same as that of traditional joint plates.

It can be seen that the failure modes of the aluminum alloy joints changed with the alteration of the planar and arch angles. It can be concluded that changes in the planar and arch angles have significant impacts on the failure modes of aluminum alloy joints.

### 6.2. Stiffness and Bearing Capacity Analysis

**Figure 18:**
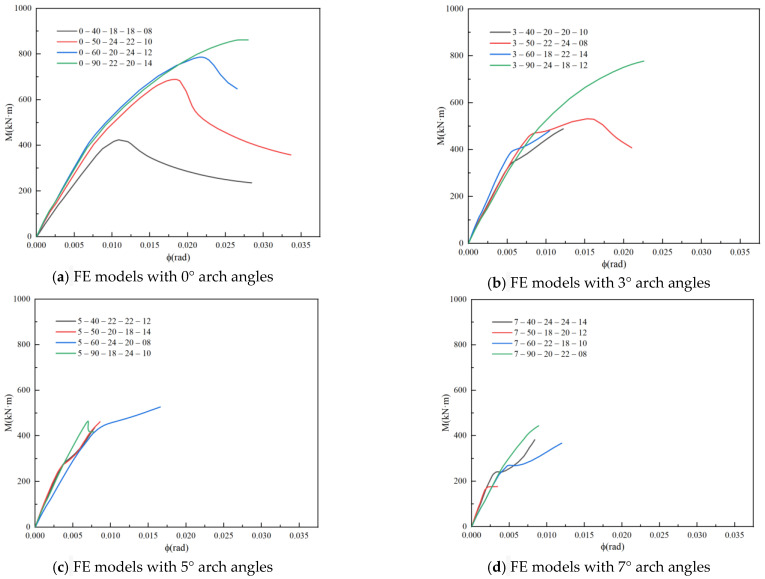
Curves of Joints.

The moment-rotation curves of the 16 groups obtained from the finite element simulation are shown in Figure 18 Because the range analysis revealed that the arch angle had the greatest impact on the initial stiffness and ultimate bearing capacity of the joints, the simulation results in this study were divided into four groups according to the different arch angles. The naming order was arch angle, joint, planar angle, plate thickness, web thickness, and flange thickness. The bending moment M of the joint can be calculated using Equation (9):

(9) $$M=F_{l}L$$

where $F_{l}$ is the vertical load applied to the joint plate, and L is the distance from the support to the load point.

**Figure 19:**
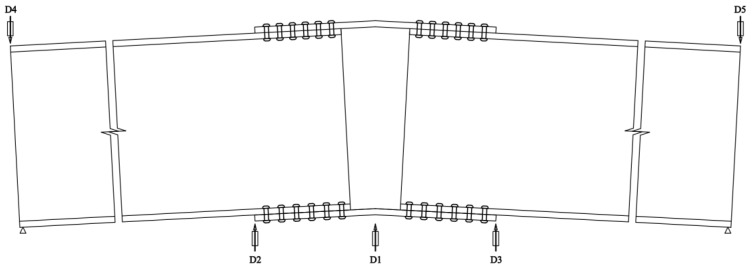
Displacement Measurement Point Schematic.

The angle calculation formula Equation (10) refers to Wang [28] and others’ calculation methods.

(10) $$\varphi =|\frac{\Delta 1-\Delta 3}{r}-\frac{\Delta 3-\Delta 5}{L_{0}}|=|\frac{\Delta 1-\Delta 2}{r}-\frac{\Delta 2-\Delta 4}{L_{0}}|$$

where Δ1–Δ5 are the corresponding displacements at measurement points 1–5, respectively, as shown in Figure 19; r is the radius of the joint plate; and $L_{0}\;$ is the distance from the edge of the joint plate to the support.

From the moment-rotation curves, it can be seen that the ultimate bearing capacity and stiffness of the different types of joints are different. Overall, there are four stages: bolt fixation, bolt slip, hole wall compression, and failure.

**Figure 20:**
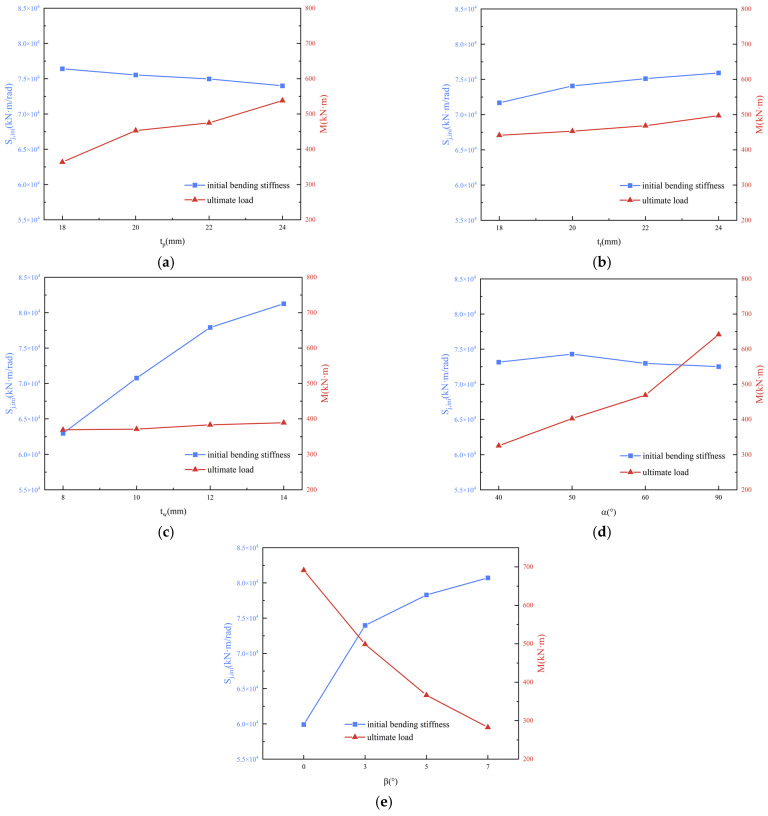
Range Analysis of Various Factors. (**a**) Effect of Gusset Plate Thickness on Initial Stiffness and Ultimate Bearing Capacity; (**b**) Effect of Flange Thickness on Initial Stiffness and Ultimate Bearing Capacity; (**c**) Effect of Web Thickness on Initial Stiffness and Ultimate Bearing Capacity; (**d**) Effect of Planar Angles on Initial Stiffness and Ultimate Bearing Capacity; (**e**) Effect of Arch Angles on Initial Stiffness and Ultimate Bearing Capacity.

In this study, the range analysis results obtained from the orthogonal experiments were also used to compare the differences between different samples and determine whether the effects of different factors (arch angles, planar angles, joint plate thickness, flange thickness, and web thickness) on the results (initial stiffness and ultimate load) were significant and their relative importance. This analysis was conducted to provide guidance for engineering applications. Figure 20 shows the results of the range analysis.

#### 6.2.1. Stiffness Analysis

The stiffness of a joint is an important parameter in evaluating its overall performance. Because aluminum alloy joints must withstand bending moments, torque, and axial forces in actual engineering, the rotational stiffness of the joint can reflect the performance of the joint under an external load. Studying the rotational performance of a joint can further determine its reliability in engineering applications. The European standard Eurocode 3 [31] specifies that the slope of the initial elastic stage of the joint moment–rotation curve should be defined as the initial rotational stiffness S_(j,ini).

As shown in Figure 20, the factors affecting the initial stiffness were significant in the following order: arch angle > web thickness > flange thickness > joint plate thickness > planar angle.

The initial stiffness of the cambered joints was greater than that of the planar joints, and the orthogonal experimental range analysis showed that the arch angle had the most significant impact on the initial stiffness of the joint. This outcome occurred mainly because the arch angles of the joint changed its stress state. Flat gusset joints are primarily subjected to bending moments and shear forces, whereas cambered gusset joints are primarily subjected to bending moments and axial forces. This fact caused the bolts to quickly contact the bolt hole wall and enter the hole–wall compression stage at the initial loading stage, thereby increasing the initial stiffness of the joint.

As shown in Figure 20a, as the joint plate thickness increases, the initial stiffness of the joint decreases slightly, mainly because, as the joint plate thickness increased, the length of the bolts also increased. The increase in length led to an increase in the stress-bearing area, whereas the cross-sectional area of the bolt and the shear-bearing capacity remained unchanged. Therefore, the stiffness decreased slightly with an increase in joint plate thickness.

Among the factors affecting the initial stiffness of aluminum alloy gusset joints, the stiffness increases with the thickness of the web and flange. However, as shown in Figure 20d, the change in the planar angle did not have a significant impact on the initial stiffness of the joint because the change in the planar angle had a minor effect on the dimensions and shapes of the bent sections, leading to a small change in the resistance moment of the section. Therefore, during the initial stage of load application, the planar angle did not affect the initial stiffness of the joint.

#### 6.2.2. Load-bearing Capacity Analysis

In this study, the ultimate load criterion was adopted to determine the load-bearing capacity for joint failure. The moment–rotation curve and range analysis revealed that the factors affecting the load-bearing capacity of the joint, in decreasing order of significance, were face angle > planar angle > gusset plate thickness > flange thickness > web thickness.

It can be observed that the change in the face angle has the most significant impact on the load-bearing capacity of the joint, followed by the change in the planar angle. Specifically, the maximum ultimate load occurred when the face angle was 0°, and the ultimate load decreased as the face angle increased. The ultimate load was maximized when the planar angle was 90° and increased as the planar angle increased. This outcome occurred primarily because the planar angle of the gusset plate altered the stress distribution and failure mode of the joint. When the planar angles between adjacent members are unequal, symmetrical sections of the gusset plate experience uneven stress, resulting in weak and strong axes. Owing to the gusset plate arching, the joint is mainly subjected to a bending moment and axial force, with the failure mode being compression failure near the weak axis in the middle of the gusset plate. When the face angle was larger, and the planar angle was smaller, the load distribution on the gusset plate became more uneven, leading to earlier compression failure in the middle of the plate and lower ultimate load-bearing capacity. In addition to the effects of the face and planar angles, the load-bearing capacity of the joint increased with the thicknesses of the gusset plate, flange, and web. However, these factors have less impact than the face and planar angles.

## 7. Conclusions

This study utilized ABAQUS modeling to analyze the effects of the planar angle, face angle, gusset plate thickness, web thickness, and flange thickness on the bending performance of a novel aluminum alloy gusset joint, and it discussed the failure modes, load transfer mechanisms, moment–rotation curves, and initial stiffness of the novel aluminum alloy arch gusset joint. The conclusions are as follows.

1. A change in the planar angle affected the stress distribution of the joint. When the angles between the members were unequal, the symmetric sections experienced uneven stress and different section moduli, resulting in strong and weak axes, respectively. The joint was more likely to rotate around the weak axis, and the weak-axis section was more susceptible to damage. When the angles between the members were equal, the symmetric sections of the gusset plate had balanced stress, and the ultimate load-bearing capacity was much greater than when the angles between the members were unequal.
2. The planar and arch angles were the main factors influencing the failure modes of the aluminum alloy gusset joints. The failure modes changed as the planar and arch angles changed. There are three main failure modes: compression failure in the middle of the gusset plate, local buckling of the web, and net cross-sectional failure of the member. Compression failure in the middle of the gusset plate occurs when adjacent members have unequal planar angles, and the gusset plate has an arching angle. The failure mode of gusset joints with a 90° angle between members and 40°, 50°, and 60° angles with 0° arch angles is local buckling of the web, and the failure mode of flat gusset joints with a 90° angle between members is the net cross-sectional failure of the member.
3. The significance of the various factors affecting the initial stiffness was ranked as follows: arch angle > web thickness > flange thickness > gusset plate thickness > planar angle. The arch angles had the most significant impact on the initial stiffness, and the initial stiffness of the gusset joints with an arching angle was greater than that of the planar gusset joints. Moreover, an increase in the gusset-plate thickness resulted in a slight decrease in the initial stiffness, whereas increases in the web and flange thicknesses led to an increase in the initial stiffness. However, changes in the planar angle did not significantly affect the initial stiffness of the gusset joints.
4. The significance of the various factors affecting the load-carrying capacity of gusset joints was ranked as follows: arch angle > planar angle > gusset plate thickness > flange thickness > web thickness. Among these factors, the arch and planar angles had the most significant effects on the load-carrying capacity of the gusset joints. This outcome occurred mainly because the arch and planar angles of the gusset plate changed the failure modes of the joints. When adjacent members had unequal planar angles, and the gusset plate was arched, the gusset plate experienced uneven stress, and the failure mode was compression failure in the middle of the gusset plate. As the thicknesses of the gusset plate, flange, and web increased, the load-carrying capacity of the gusset joints increased.
5. Based on the research analysis of the study results, we can make the following recommendations for practical engineering: (1) Changes in the planar angle will affect the stress distribution of the joint, causing the joint to enter the plastic phase prematurely, with the primary failure mode being the mid-section crushing the joint plate, which does not align with the “strong joint, weak member” concept. In real-world engineering, we should avoid this situation as much as possible by minimizing the inconsistency in the angles between members; (2) The initial stiffness of the joint will increase with an increase in the planar angle, but in cases in which the angles between the members are not equal, an increase in the planar angle will lead to a rapid decrease in the ultimate bearing capacity. In practical engineering, we recommend setting the planar angle equal to the angles between the members (all angles between the members are either 60° or 90°) to maximize the joint’s load-bearing performance.

## Figures and Tables

**Table 1:** Orthogonal Experiment Table.

Number	Arch Angles	Planar Angles	Thickness of Joint Plate	Thickness of Flange	Thickness of Web
	(°)	(°)	(mm)	(mm)	(mm)
1	0	40	18	18	8
2	0	50	24	22	10
3	0	60	20	24	12
4	0	90	22	20	14
5	3	40	20	20	10
6	3	50	22	24	8
7	3	60	18	22	14
8	3	90	24	18	12
9	5	40	22	22	12
10	5	50	20	18	14
11	5	60	24	20	8
12	5	90	18	24	10
13	7	40	24	24	14
14	7	50	18	20	12
15	7	60	22	18	10
16	7	90	20	22	8

**Table 2:** Material Properties of Components.

Component	Material	ρ (kg/m^3^)	E (MPa)	ν	f_0.2_ (MPa)	f_u_ (MPa)	f_v_ (MPa)
H-shaped members; Joint plates	6061T6	2700	70,400	0.33	264	304	180
Bolt	304HC	7850	189,000	0.3	460	720	504

**Table 3:** Characteristics of Contact Pairs.

Contact or Constraint	Master Surface	Slave Surface	Tangential Behavior	Friction Coefficient	Normal Behavior
Bolts-to-plate	Bolts	Plate	Penalty	0.3	Hard contact
Nuts-to-member flange	Nuts	Flange	Penalty	0.3	Hard contact
Member flange-to-plate	Plate	Flange	Penalty	0.3	Hard contact
Bolt shanks-to-bolt holes	Bolt shanks	Bolt holes	Frictionless	None	Hard contact

## Data Availability

Data will be made available on request.
